# Preparation of γ-TiAl Alloys via Self-Propagating Aluminothermic Reduction–Slag Washing Refining Coupled with Vacuum Arc Remelting

**DOI:** 10.3390/ma19081650

**Published:** 2026-04-20

**Authors:** Han Jiang, Ting-An Zhang, Zhi-He Dou

**Affiliations:** 1 School of Metallurgy, Northeastern University, Shenyang 110819, China; jiangh14@mails.neu.edu.cn; 2 Key Laboratory of Ecological Metallurgy of Multi-Metal Intergrown Ores of the Ministry of Education, Shenyang 110819, China

**Keywords:** γ-TiAl intermetallic compound, self-propagating high-temperature synthesis, slag washing refining, vacuum arc remelting, impurity removal, inclusion refinement

## Abstract

Conventional titanium alloy production based on the Kroll process features high energy consumption and long procedures, making low-cost, short-process fabrication a research focus in titanium metallurgy. In this work, low-interstitial γ-TiAl alloys were prepared via a coupled self-propagating high-temperature synthesis (SHS)–slag washing refining–vacuum arc remelting (VAR) process using TiO_2_ as the raw material. Slag washing refining was performed at 1750 °C with 150 g of CaO-Al_2_O_3_-SiO_2_-CaF_2_ mold flux and 1.5 wt.% Ca, followed by VAR under a vacuum of 10^−2^–10^−3^ Pa. γ-TiAl alloy with a composition of Ti 66.01 ± 0.5 wt.%, Al 33.8 ± 0.5 wt.%, O 0.054 ± 0.002 wt.%, N 0.046 ± 0.005 wt.%, and C 0.085 ± 0.008 wt.% was obtained, and the inclusion size was refined to 0–3 μm. This coupled approach provides a scalable, low-cost route for the industrial preparation of low-interstitial γ-TiAl alloys.

## 1. Introduction

γ-TiAl intermetallic compounds are considered key next-generation high-temperature structural materials for aerospace and automotive engine applications owing to their low density, high specific strength, excellent high-temperature oxidation resistance, and creep resistance [1,2,3,4,5]. However, large-scale engineering applications of γ-TiAl alloys are severely restricted by the traditional powder metallurgy process, which suffers from high production costs, complex procedures, and long preparation cycles [6,7,8].

SHS has been widely applied in the preparation of TiAl alloys and their precursors owing to its advantages, such as fast reaction rates, low energy consumption, and short process flow [9]. This technology achieves atomic-level homogeneous synthesis through the highly exothermic aluminothermic reduction reaction, which can significantly shorten the preparation process and reduce energy input. Nevertheless, γ-TiAl alloys prepared via direct SHS usually exhibit high oxygen content, large inclusion size, and inhomogeneous microstructure [10], mainly caused by insufficient atmospheric control during the SHS reaction and inadequate slag–metal separation due to the excessively fast cooling rate [11,12].

At present, the mainstream preparation technologies for TiAl alloys include powder metallurgy [13,14,15,16,17,18], melting and casting [19], cold spray–hot isostatic pressing composite technology [20], and laser powder bed fusion (L-PBF) additive manufacturing [21,22]. Among them, VAR is a standard process for fabricating high-purity titanium-based alloys [23,24,25,26], as it can effectively remove volatile impurities and realize composition homogenization via high-temperature remelting. These advantages make VAR an ideal complement to the SHS process, overcoming the drawbacks of high impurity content and inhomogeneous structure in SHS primary ingots.

Previous research on SHS-based TiAl synthesis primarily focused on verifying reaction feasibility at the laboratory scale, typically yielding intermediate precursors with high oxygen content (1.0–2.6%) [10,11,12]. This work achieves a significant scale-up to the kilogram level, or even tens of kilograms per batch, and shifts the research focus toward a ‘SHS–Slag Washing–VAR’ thermochemical coupling mechanism. By leveraging the complementary advantages of active slag deoxidation and vacuum arc denitrification, a ‘relay-style’ impurity regulation is established. This approach successfully produces high-performance, low-interstitial γ-TiAl structural alloys directly from oxides, significantly enhancing production yields while reducing costs.

## 2. Experimental

### 2.1. Raw Materials

Most of the raw materials used in the experiment were of analytical grade, while the titanium dioxide (TiO_2_) and aluminum (Al) particles during the SHS process were of industrial grade. High-purity argon (99.99 vol.%) was used as the protective gas. The detailed specifications and manufacturers of all reagents are listed in Table 1. In the SHS reaction, titanium dioxide and aluminum particles were used as the main reactants, potassium chlorate (KClO_3_) was used as the exothermic agent, and calcium oxide (CaO) was used as the slag former. In the medium-frequency induction slag washing refining process, calcium (Ca) particles were used as the secondary deoxidizer, and a quaternary CaO-Al_2_O_3_-CaF_2_-SiO_2_ mold flux was adopted to adjust the physicochemical properties of the molten pool.

### 2.2. Experimental Procedure

**Figure 1:**
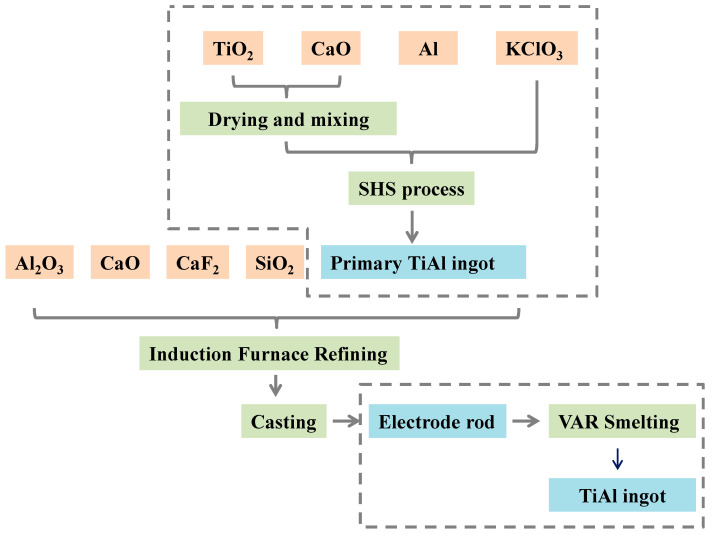
Schematic diagram of the process flow.

Figure 1 shows a schematic diagram of the entire preparation process of γ-TiAl alloys via SHS–slag washing refining coupled with VAR. The specific experimental steps were as follows:

1. SHS process: Raw materials were dried, weighed, and uniformly mixed according to the ratios listed in Table 2. The mixture was loaded into a graphite crucible reactor, and the SHS reaction was ignited with a small amount of magnesium powder. During the reaction, the remaining raw materials were continuously fed to maintain a stable and complete reaction. After naturally cooling to room temperature, slag–metal separation was performed to obtain the primary SHS TiAl alloy ingot.
2. Slag washing refining process: The primary SHS ingot was crushed and placed in a graphite crucible inside an induction furnace. Designed proportions of calcium aluminate-based mold flux and Ca particles (relative to the alloy mass) were added, and high-purity Ar gas was continuously introduced as a protective atmosphere. The mixture was inductively heated until the slag and metal were fully melted, and then held at the target temperature for 5 min to complete sufficient refining. The melt was rapidly cast to fabricate TiAl consumable electrodes suitable for subsequent VAR.
3. VAR melting process: The as-cast electrode after slag washing refining was mounted on an electrode holder in a VAR furnace. The chamber was evacuated to a pressure of 10^−2^–10^−3^ Pa, and the arc was ignited to conduct steady and uniform remelting until the electrode was completely melted. For a 5 kg charge, the melting duration was approximately 8–10 min, with a melting current of 600–900 A and a melting voltage of 28–38 V. The ingot was fully cooled in a water-cooled copper crucible before being extracted, yielding the final γ-TiAl alloy ingot with low inclusion content.

**Figure 2:**
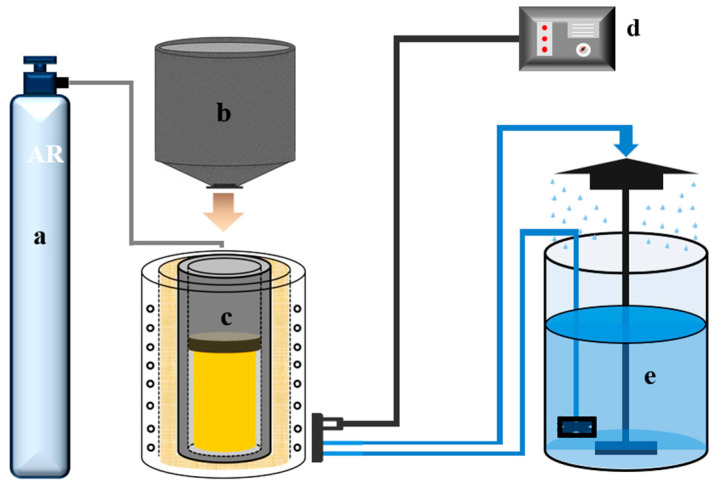
Schematic diagram of slag washing refining equipment: (**a**) argon; (**b**) SHS reactor; (**c**) medium-frequency induction furnace; (**d**) control power supply; (**e**) water cooling tower.

A schematic diagram of the slag washing refining equipment is shown in Figure 2. The material ratio during the SHS process is shown in Table 2.

### 2.3. Characterization and Testing Methods

Phase composition analysis: An X-ray diffractometer (XRD, Bruker D8 ADVANCE, Karlsruhe, Germany) with Cu Kα radiation (λ = 1.5406 Å) was used for phase characterization, with a scan rate of 4 °·min^−1^ and a 2θ range of 10–90°. Chemical composition analysis: An inductively coupled plasma optical emission spectroscopy (ICP-OES, Thermo Fisher Scientific, Waltham, MA, USA) and oxygen-nitrogen analyzer (LECO ONH836, St. Joseph, MI, USA) were used to determine the contents of main elements and interstitial impurity elements in the alloy, respectively [27]. Microstructural characterization: A scanning electron microscope (SEM, Zeiss Sigma 300, Oberkochen, Germany) was used to observe the microstructural morphologies and inclusion distributions of the alloy, with an accelerating voltage of 20 kV and a beam current of 20 mA. An energy-dispersive spectroscope (EDS, Oxford Instruments, Abingdon, UK) was attached to the SEM to perform elemental composition analysis at the microscale.

To ensure the statistical reliability and reproducibility of the experimental data, all key quantitative measurements—including chemical composition analyses (ICP-OES and LECO) and compressive mechanical tests—were performed using at least three independent replicates (*n* ≥ 3) under identical conditions. The quantitative results are expressed as the mean value ± standard deviation (SD).

## 3. Results and Discussion

### 3.1. Thermodynamic Design and Feasibility Verification

To screen the optimal mold flux for slag washing refining of γ-TiAl alloys, the multicomponent phase diagrams of the CaO-Al_2_O_3_, CaO-Al_2_O_3_-CaF_2_, CaO-Al_2_O_3_-CaF_2_-SiO_2_, and CaO-Al_2_O_3_-CaF_2_-MgO slag systems were drawn using FactSage 8.3 thermodynamic software to study their melting temperature changes and phase compositions [28,29].

**Figure 3:**
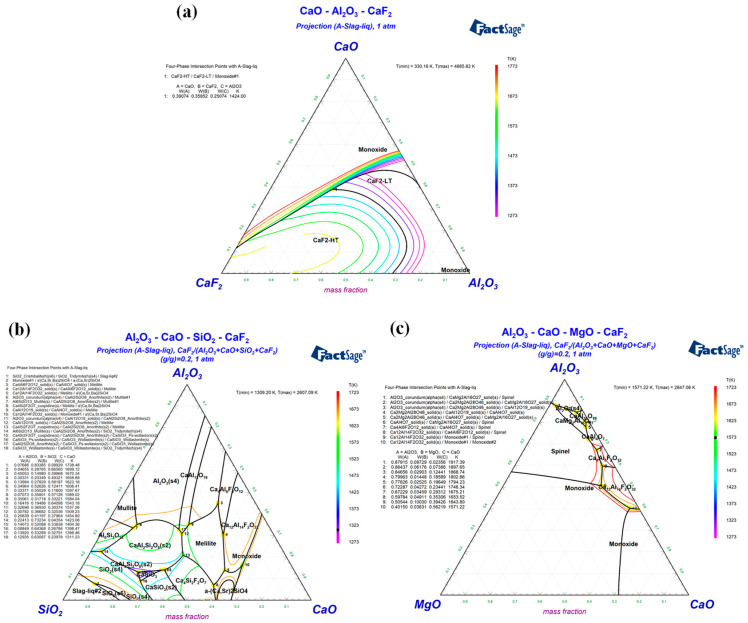
Multicomponent phase diagram of the slag systems: (**a**) CaO-Al_2_O_3_-CaF_2_; (**b**) CaO-Al_2_O_3_-SiO_2_-CaF_2_; (**c**) CaO-Al_2_O_3_-MgO-CaF_2_.

Figure 3a shows the ternary phase diagram of the CaO-Al_2_O_3_-CaF_2_ slag system, which mainly includes the CaF_2_-HT and CaF_2_-LT regions. The low-melting-temperature region is concentrated in the area with a CaF_2_ content of less than 25 wt.%, so the CaF_2_ content in the mold flux was selected to be less than 30 wt.%. Figure 3b presents the quaternary phase diagram of CaO-Al_2_O_3_-SiO_2_-CaF_2_, and the main phases are CaAl_2_Si_2_O_8_, CaSiO_3_, Ca_4_Si_2_F_2_O_7_, and Ca_4_Al_6_F_2_O_12_. The minimum melting temperature of this slag system is 1311.23 K, and the low-melting-temperature region is distributed in the area with CaO < 60 wt.% and Al_2_O_3_ < 40 wt.%. Figure 3c displays the quaternary phase diagram of CaO-Al_2_O_3_-MgO-CaF_2_, with the main phases of CaAl_4_O_7_, CaAl_12_O_19_, and CaMg_2_Al_16_O_27_. This slag system has a relatively high melting temperature (minimum 1571.22 K), and the low-melting-temperature region is concentrated in the area with CaO > 50 wt.% and Al_2_O_3_ < 50 wt.%.

The slag density was calculated using the additive model proposed by Bottinga et al. [25], and the basic calculation formulas are as follows:

(1) $$\rho =\frac{\sum_{i}\;\;X_{i}M_{i}}{V}$$

(2) $$V=\sum_{i}\;X_{i}{\bar{V}}_{i}$$

where *V* is the molar volume of the slag (cm^3^·mol^−1^); ρ is the slag density (g·cm^−3^); *X_i_* and *M_i_* are the mole fraction and molar mass of component *i* (g·mol^−1^), respectively; and ${\bar{V}}_{i}$ is the partial molar volume of component *i* (cm^3^·mol^−1^).

Taking the CaO-Al_2_O_3_-CaF_2_ ternary slag system as an example, the slag density *ρ_p_* was calculated using the molar volume *V_m_*:

(3) $$\rho \;=\;\frac{\sum \left(x_{i}M_{i}\right)}{\sum \left(x_{i}V_{i}\right)\cdot \left[1+\alpha \left(T-T_{\mathrm{ref}}\right)\right]}$$

where *V_m_* is the molar volume of the slag (cm^3^·mol^−1^), and the partial molar volume of each component is assumed to be equal to that of the pure substance.

The volume of oxide inclusions is mainly affected by the temperature and composition, and there is an approximately linear relationship between the molar volume *V_m_* and temperature *T*, as shown in Equation (4):

(4) $$\begin{array}{c} V=\sum_{i}X_{i}V_{i}+\alpha \left(T-T_{\mathrm{ref}}\right)\\ =X_{{\mathrm{Al}}_{2}O_{3}}V_{{\mathrm{Al}}_{2}O_{3}}+X_{{\mathrm{CaF}}_{2}}V_{{\mathrm{CaF}}_{2}}+X_{\mathrm{CaO}}V_{\mathrm{CaO}}+\alpha \left(T-T_{\mathrm{ref}}\right) \end{array}$$

where *V_i_* is the partial molar volume of component *i* at the reference temperature (cm^3^·mol^−1^); *α* is the thermal expansion coefficient (cm^3^·(mol·K)^−1^); *T_ref_* is the reference temperature (1773 K, 1500 °C); and *T* is the absolute temperature (K). The unknown parameters *V_i_* and *α* were optimized and solved using the least squares method in MATLAB R2023b (Version 23.2), and the fitting results are listed in Table 3. The reference temperature was set to 1773 K according to the actual smelting temperature of TiAl alloys (1400–1800 °C) and the literature [25].

Based on the study by Shi et al. [30], who investigated slag viscosity using the internal rotating cylinder method with an RTW-10 melt property tester, the viscosity characteristics of different slag systems were obtained: (1) the viscosity of the CaO-Al_2_O_3_ binary slag system is the lowest when the CaO content is 30 wt.%; (2) the viscosity and turning point temperature of the CaO-Al_2_O_3_-CaF_2_ ternary slag system in the high-temperature region decrease with the increase in CaF_2_ content; (3) the viscosity and turning point temperature of the CaO-Al_2_O_3_-MgO ternary slag system in the high-temperature region first decrease and then increase with the increase in MgO content, reaching the minimum at 3 wt.% MgO; and (4) the viscosity and turning point temperature of the CaO-Al_2_O_3_-SiO_2_ ternary slag system in the high-temperature region increase with the increase in SiO_2_ content.

**Figure 4:**
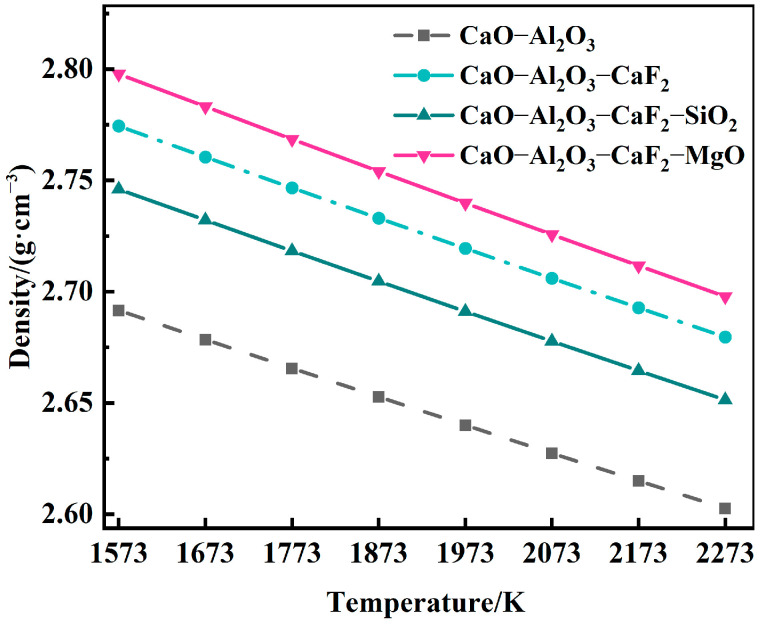
Trend diagram of density variation of four slag types with temperature.

Considering the melting point and viscosity of the slag systems comprehensively, the densities of four slag types with different mass ratios (CaO:Al_2_O_3_ = 1:2; CaO:Al_2_O_3_:CaF_2_ = 0.27:0.53:0.2; CaO:Al_2_O_3_:CaF_2_:SiO_2_ = 0.247:0.493:0.2:0.06; and CaO:Al_2_O_3_:CaF_2_:MgO = 0.34:0.39:0.2:0.07) were calculated, and the results are shown in Figure 4.

The CaO-Al_2_O_3_ binary slag system has the lowest density but the highest melting point [10], followed by the CaO-Al_2_O_3_-CaF_2_-SiO_2_ quaternary slag system with the lowest melting point, and the CaO-Al_2_O_3_-CaF_2_-MgO quaternary slag system has the maximum density. Based on the comprehensive analysis of density and melting point, the CaO-Al_2_O_3_-CaF_2_-SiO_2_ quaternary slag system was selected as the optimal mold flux for the slag washing refining of γ-TiAl alloys due to its low melting point and suitable density. Finally, this study selected the mold flux ratio as CaO:Al_2_O_3_:CaF_2_:SiO_2_ = 0.247:0.493:0.2:0.06. Under these conditions, the optimized mold flux exhibits a low melting point, good fluidity, and introduces a low level of interstitial impurity elements.

**Figure 5:**
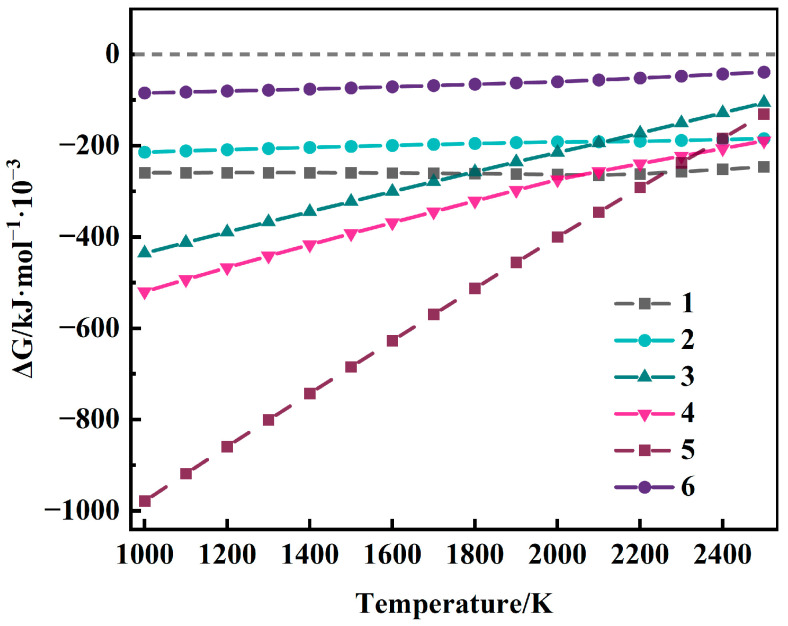
ΔG curves of possible reactions between titanium oxide and Ca.

The SHS-derived primary TiAl ingots contain low-valence titanium oxides such as Ti_3_O_5_ and TiO [10], which need to be deeply deoxidized by adding Ca during the isothermal holding stage. The possible reactions between Ca and the main titanium oxides (TiO_2_, Ti_3_O_5_, and TiO) are listed in Table 4. The Gibbs free energy (Δ*G*) of each reaction was sourced from the FactSage 8.3 database, and the Δ*G–T* curves were drawn (Figure 5).

As shown in Figure 5, the ΔG values of all reactions are less than 0 in the temperature range of 1000–2200 K [31,32,33], indicating that the reduction of titanium oxides by Ca can proceed spontaneously, which verifies the thermodynamic feasibility of Ca as an efficient deoxidizer for γ-TiAl alloys. Meanwhile, the ΔG values of all reactions increase with the increase in temperature, which means that high temperatures are unfavorable for the deoxidation reaction. In addition, the ΔG values increase with decreasing valence of titanium oxides, indicating that the reduction to metallic titanium becomes more difficult as the oxide valence decreases. The experiments were performed under Ar protection to minimize the oxygen partial pressure, which theoretically supports the secondary reduction of oxides by Ca in the melt at high temperatures after the molten pool forms.

**Figure 6:**
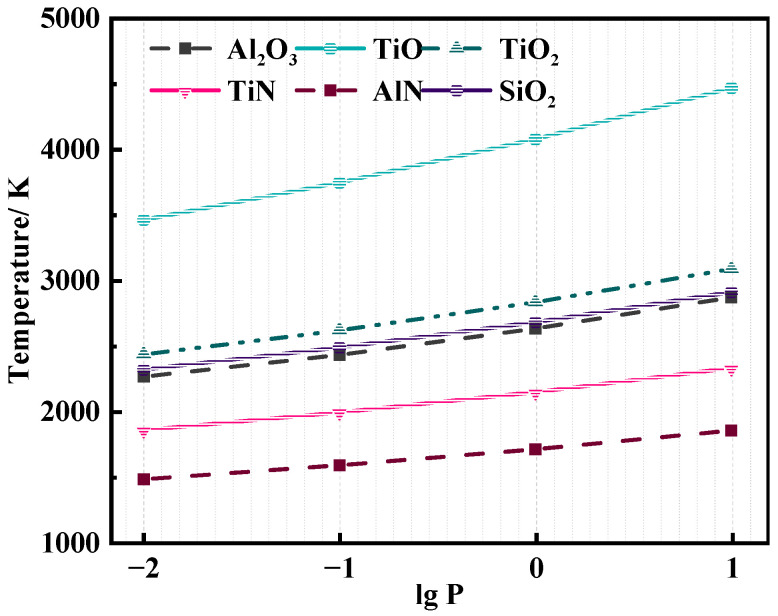
Thermal decomposition temperature of nitrides and oxides under different vacuum degrees.

The main nitrides in the SHS primary ingots are TiN and AlN, which need to be removed through the subsequent VAR process. The thermal decomposition reactions of the main oxides and nitrides in the alloy are listed in Table 5, and their phase–temperature (P–T) diagrams under different pressures are shown in Figure 6.

It can be seen from Figure 6 that, in the temperature range of 1000–5000 K, the decomposition temperature of nitrides is significantly lower than that of oxides at the same pressure. The decomposition temperature of the same substance decreases with the decrease in system pressure, indicating that the high temperature and high vacuum (low pressure) environment is beneficial for the decomposition of inclusions.

The temperature of the molten pool in the copper crucible during the VAR process is basically in the range of 1600–1800 K. AlN and TiN can be effectively removed via thermal decomposition into simple substances under a vacuum of 10^−3^–10^−2^ Pa, while the decomposition temperature of oxides is generally above 2300 K, which is not reached during the smelting process. Therefore, the oxides are removed via arc-induced fragmentation, floatation to the slag–metal interface, and adsorption by the mold flux instead of thermal decomposition.

Based on the standard thermal decomposition reactions of interstitial nitrides in titanium alloy systems [34], the decomposition processes of AlN and TiN inclusions under high-temperature vacuum conditions are described by Equations (5) and (6):

AlN→Al + 1/2N_2_(g) (5)

TiN→Ti + 1/2N_2_(g) (6)

**Figure 7:**
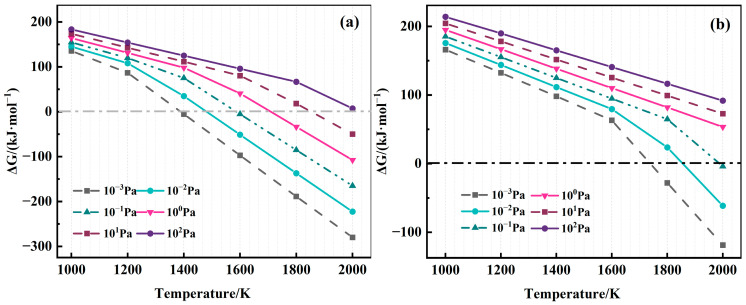
ΔG–T diagrams of AlN and TiN decomposition: (**a**) AlN; (**b**) TiN.

Figure 7 shows the Δ*G–T* curves for the thermal decomposition of AlN and TiN. The data is sourced from FactSage 8.3. It can be seen that the Δ*G* value of the AlN decomposition reaction is less than 0 when the temperature exceeds 1600 K and the chamber pressure is lower than 10^−1^ Pa, which meets the thermal decomposition conditions. In the VAR smelting temperature range of 1600–2700 K, the furnace pressure needs to be maintained below 10^−1^ Pa to ensure the complete decomposition of AlN. An increase in temperature can enhance the vacuum effect of the furnace and promote the removal of AlN from the metal matrix. By contrast, the decomposition of TiN requires a temperature higher than 1800 K and a pressure lower than 10^−2^ Pa. Although the arc temperature during the VAR process can reach 2000–2700 K, which meets the TiN decomposition conditions for instantaneous contact, TiN removal may be incomplete because the arc-contacted metal melts rapidly and drips into the molten pool at a fast melting rate. Therefore, the VAR process can effectively remove N from the alloy, but residual inclusions may appear if the vacuum pressure fails to meet the impurity decomposition conditions.

### 3.2. Phase and Composition Evolution During the Process

**Figure 8:**
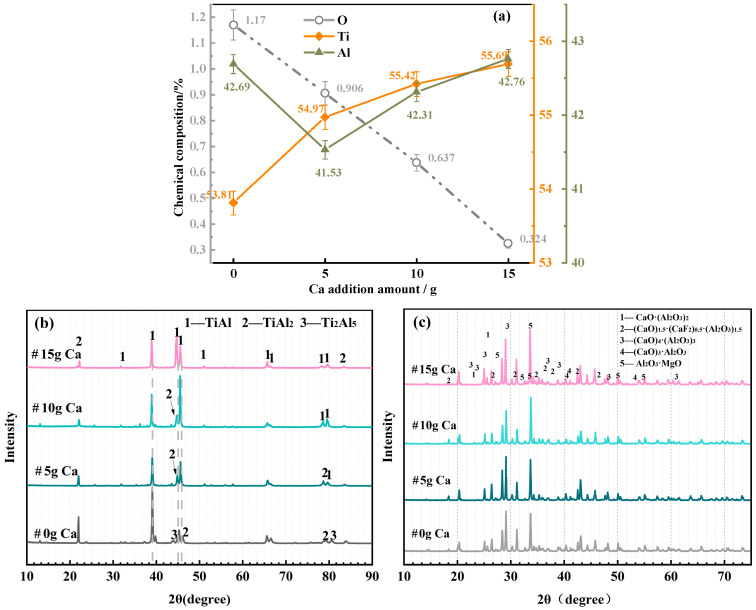
The effects of different calcium additions on (**a**) alloy chemical composition, (**b**) alloy phase, and (**c**) slag system phase.

The main composition and phase evolution of the alloy during the coupled process were investigated using 1000 g of alloy raw material as the standard, and the effects of different Ca additions on the alloy chemical composition, alloy phase composition, and slag phase composition are shown in Figure 8.

Figure 8a shows the effect of different Ca additions on the key elements of the alloy. The oxides in the alloy exist in the form of Al_2_O_3_ inclusions and titanium oxides with different valences (TiO_x_). Without Ca addition, the large oxide inclusions in the fully molten metal are adsorbed by the mold flux, while Al_2_O_3_ and TiO_x_ inclusions of less than 5 μm are free in the alloy melt and remain in the metal after cooling. With the increase in Ca addition, the O content in the alloy decreases continuously, indicating that Ca has a significant deoxidation effect. The deoxidation effect is remarkable in the range of 0–10 g Ca addition (per 1000 g alloy raw material), and the O content tends to be stable with further increase in Ca addition. This is because Ca can reduce TiO_x_ to metallic Ti at high temperatures, leading to a slow increase in the Ti content in the alloy. Once the reduction reaction reaches equilibrium, the content of each element stabilizes, and the excess Ca volatilizes in the form of vapor or floats to the slag surface, which has no effect on the effective components of the alloy.

As shown in Figure 8b, the main phase of the alloy after deep deoxidation is still the γ phase, and the diffraction intensities of the TiAl_2_ and Ti_2_Al_5_ phases decrease. With the increase in Ca addition, the alloy phases transform into Al-lean phases, which is due to the enhanced reduction reaction with the increase in Ca addition, leading to an increase in the Ti content in the alloy matrix and the enhancement of the γ-phase diffraction peak in the XRD pattern.

Figure 8c displays the effect of different Ca additions on the slag phase composition. The main components of the slag are binary or ternary composite phases composed of CaO, CaF_2_, Al_2_O_3_, and MgO. The diffraction peak intensities of the (CaO)_4_·(Al_2_O_3_)_3_ and (CaO)_3_·Al_2_O_3_ phases increase gradually with the increase in Ca addition during the deep reduction process. This is because Ca reacts with O in the alloy melt to form CaO at high temperatures, and the excess Ca volatilizes or floats to the slag surface, where it rapidly oxidizes into CaO upon contact with residual oxygen, resulting in an increase in the proportion of Ca-rich phases in the slag.

**Figure 9:**
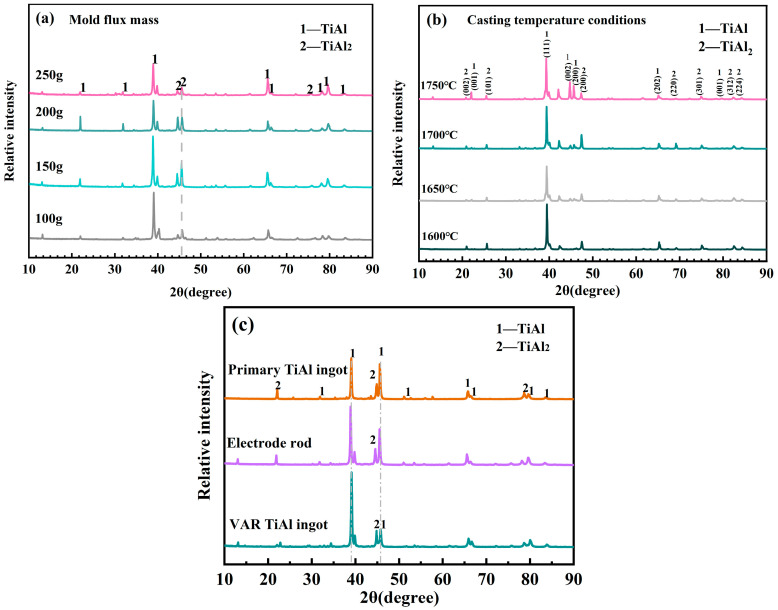
XRD patterns of the alloy (**a**) under different mold flux conditions during slag washing refining, (**b**) under different casting temperature conditions during slag washing refining, and (**c**) comparison of the entire process.

Figure 9 shows the XRD patterns of the alloy under different process conditions, including different mold flux additions, different casting temperatures, and process comparisons. As shown in Figure 9a, the main phases of the alloy are the TiAl and TiAl_2_ phases under different mold flux additions, and no new phases are formed with the change in slag dosage. The crystallization quality of the cast products with 100 g and 150 g mold flux additions (per 1000 g alloy raw material) is better than that with 200 g and 250 g additions. The reason is that an increase in mold flux addition raises the proportion of slag phase in the melt, and the excess slag phase fails to precipitate in time and consolidates in the matrix during the rapid solidification process, which hinders grain growth.

Figure 9b shows that the TiAl_2_ phase gradually precipitates in the matrix with the increase in casting temperature, and its diffraction peak intensity increases with the increase in temperature. An increase in casting temperature promotes the diffusion of more O, N, C, and other elements into the matrix, and these elements have extremely low solid solubility in the γ-TiAl lattice [35]. Most of them combine with Ti atoms to form nitrides and oxides, resulting in the local enrichment of Al atoms and thus the precipitation of the TiAl_2_ phase.

Figure 9c compares the alloy XRD patterns before and after the coupled process. The SHS primary TiAl ingots have a high content of the TiAl_2_ phase, which decreases gradually after slag washing refining and VAR treatment. The TiAl ingots obtained after VAR have the highest TiAl phase intensity and the purest phase composition. Therefore, the Al content in the alloy decreases continuously during the coupled process, which is mainly caused by the volatilization of Al at high temperature [36].

### 3.3. Improvements in Impurity Elements and Inclusions by the Process

**Figure 10:**
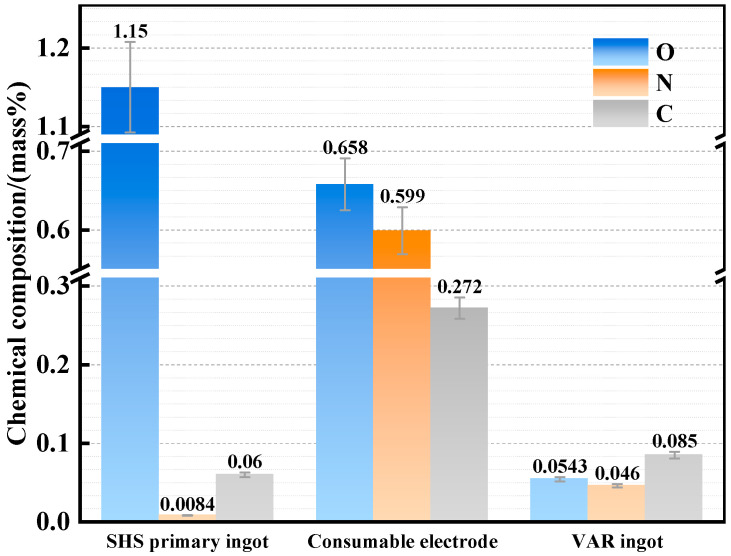
Contents of interstitial impurity elements in the alloy at different process stages.

Figure 10 shows the improvement in impurity element contents in the alloy at different process stages (SHS primary ingots, cast consumable electrodes, and VAR products). Through slag washing refining and VAR treatment, the O content in the alloy is reduced from 1.15 wt.% to 0.054 wt.%; the increased N content during the casting process is also significantly reduced through the subsequent VAR process; and the C content in the final alloy is reduced to 0.085 wt.%. Because the SHS process isolates the melt from the air but involves oxygen-rich raw materials, the primary ingot exhibits a high oxygen content and an extremely low nitrogen content. By contrast, the slag washing refining process involves instantaneous contact with the air during casting and high-temperature melt exposure to the graphite crucible, leading to a significant increase in N content and carburization. Finally, the impurity element contents are sufficiently reduced under the vacuum conditions of VAR.

This indicates that the coupled SHS–slag washing refining–VAR process has a significant effect on the deep removal of oxide inclusions in the γ-TiAl alloy.

**Figure 11:**
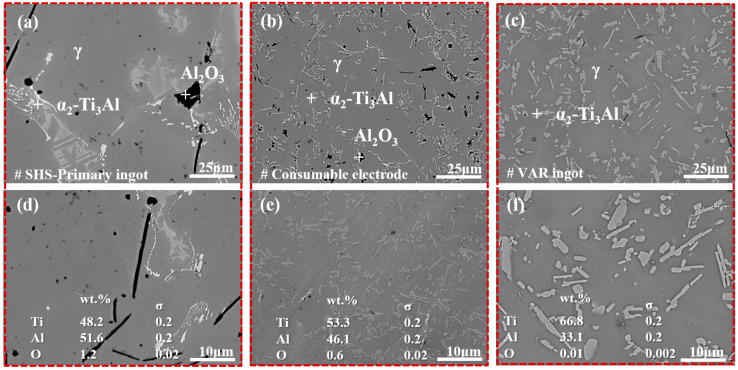
Comparison of micro-morphologies at different process stages: (**a**,**d**) SHS primary ingot; (**b**,**e**) consumable electrode; (**c**,**f**) alloy ingot after VAR.

Figure 11 shows the microstructural morphologies of the alloy at different process stages. The second phase in the SHS primary ingots is the α_2_ phase (the bright area in Figure 11a,d), which is mostly distributed in the form of massive and dot-like aggregates. Meanwhile, the α_2_ phase and γ phase form a lamellar structure, and the inclusions are large and distributed in the form of long strips and large blocks. After slag washing refining, the grains of the consumable electrode are significantly refined and uniformly dispersed in the matrix phase (Figure 11b,e). The inclusion size is reduced to 5–10 μm, and the inclusions transform from large blocks/long strips into fine particles with random distribution. In the alloy ingots after VAR treatment (Figure 11c,f), the second phase is mostly the lath-shaped α_2_ phase, which is uniformly distributed in the matrix without aggregation. At the same time, the inclusions are effectively removed, and the inclusion size is mostly less than 3 μm.

**Figure 12:**
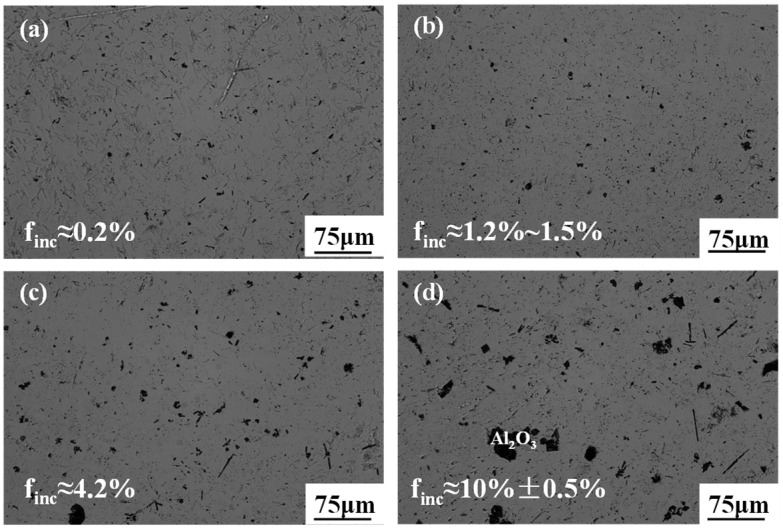
Metallographic microanalysis of alloys with the different mass of mold flux: (**a**) 100 g; (**b**) 150 g; (**c**) 200 g; (**d**) 250 g. (Note: *f_inc_* represents the estimated area fraction of inclusions).

Figure 12 shows metallographic micrographs of the alloy with different mold flux additions during the slag washing refining process. To robustly evaluate the microstructural evolution, the area fraction of the residual slag inclusions (*f_inc_*) was systematically estimated. Inclusions are randomly distributed in the matrix, and the residual amount of the slag phase increases significantly with higher mold flux additions. When the mold flux addition is ≤150 g (per 1000 g alloy raw material), the metal structure is highly dense, with the inclusion size restricted to 0–20 μm. As shown in Figure 12a,b, the inclusion area fraction remains extremely low (*f_inc_ ≈* 0.2% to 1.5%). However, when the mold flux addition reaches 200 g and above, the inclusions become much more densely distributed, and their size dramatically increases up to 0–75 μm. The quantitative estimation reveals a significant surge in the residual slag fraction to *f_inc_ ≈* 4.2% (Figure 12c), peaking at roughly 10 ± 0.5% at excessive dosages (Figure 12d). Furthermore, localized EDS point analysis (as marked in Figure 12d) explicitly confirmed that these massive blocky residues are predominantly Al_2_O_3_-based entrapped slag. This severe slag entrapment occurs because the increased proportion of the liquid slag phase in the melt impedes its timely floatation and separation, causing excess slag to remain trapped in the matrix during rapid cooling and solidification. Ultimately, while the mold flux addition does not alter the fundamental phase composition, optimizing the dosage (e.g., ≤150 g) is crucial for minimizing macroscopic inclusion defects and ensuring high crystallization quality by effectively regulating O and N impurities.

**Figure 13:**
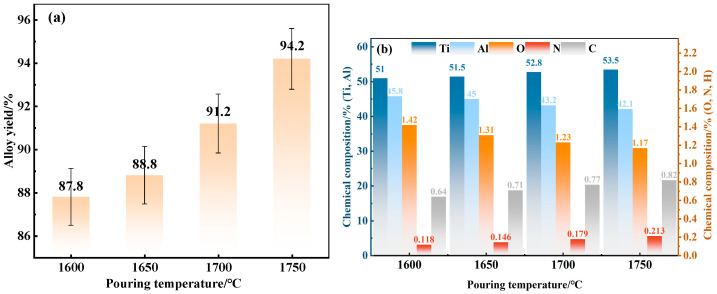
Yield and chemical composition of the alloy at different casting temperatures: (**a**) alloy yield; (**b**) chemical composition of alloying elements.

Figure 13 shows the alloy yield and chemical composition at different casting temperatures during the slag washing refining process. Controlling the casting temperature can effectively improve the impurity element contents in the alloy. Combined with the previous conclusion that the Al content is negatively correlated with the casting temperature, the O and Al contents in the alloy decrease with the increase in casting temperature, while the C, N, and Ti contents increase (Figure 13b). This is because high temperatures promote the further floatation of inclusions in the alloy: Al volatilizes at elevated temperatures, while C and N are more likely to form compounds with metal elements at high temperatures. The alloy yield first increases and then decreases with the increase in casting temperature (Figure 13a), and the optimal pouring temperature is determined to be 1750 °C. Although the contents of C and N increase slightly at this temperature, the alloy yield is the highest. Strictly controlling the holding time at 5 min and repeating the experiment yields a casting yield error within 1.5%.

**Figure 14:**
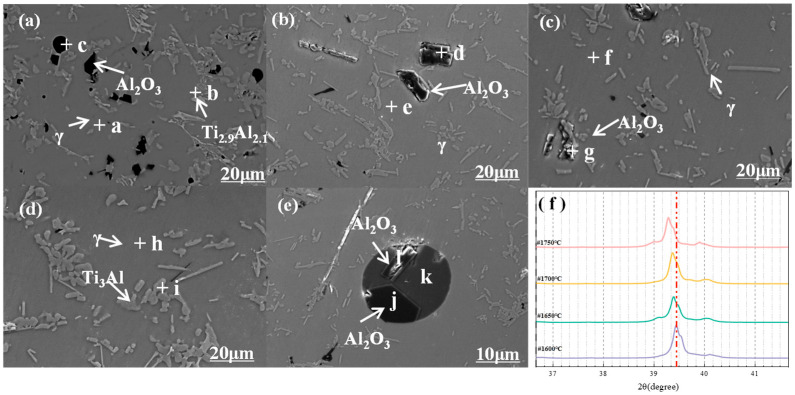
SEM images at different casting temperatures: (**a**) 1600 °C; (**b**) 1650 °C; (**c**) 1700 °C; (**d**) 1750 °C; (**e**) inclusion distribution; (**f**) main peak shift.

Figure 14 shows SEM images of the alloy at different casting temperatures. The EDS results of the marked points are shown in Table 6. The alloy samples are composed of the metal matrix phase, spherical or lath-shaped second phase, and inclusions at different casting temperatures. The inclusions are irregular in shape with a size of 0–15 μm, and they are identified as Al_2_O_3_ via EDS analysis. The number of inclusions decreases with the increase in casting temperature, and the inclusion size is less than 5 μm with random distribution in the matrix when the temperature reaches 1750 °C (Figure 14d). The Al content in the matrix decreases with the increase in casting temperature, and the distribution of the Ti_3_Al phase increases. The compactness and microstructural uniformity of the alloy are improved with the increase in casting temperature, which is because the high temperature reduces the viscosity and improves the fluidity of the slag in the molten metal, thus enhancing the slag–metal separation effect, which is beneficial for the homogenization of the alloy matrix and the reduction of Al_2_O_3_ inclusion size.

Figure 14e shows an SEM image illustrating the morphology of entrapped slag particles in the alloy. The slag phase exhibits distinct features: its inclusion size ranges from 30 to 100 μm, which is much larger than Al_2_O_3_ inclusions; they appear as regular spherical particles with clear outlines and sharp interfaces with the matrix; and its volume fraction is low. Such spherical slag inclusions form because the metal melt adjacent to the slag solidifies preferentially during casting, leaving part of the mold flux trapped inside the matrix without sufficient flotation. The slag phase in Figure 14e is confirmed as CaO·Al_2_O_3_. The Al_2_O_3_ phase encapsulated in the slag is angular and regularly polygonal, and it is distinct from the irregular Al_2_O_3_ in the matrix, indicating that Al_2_O_3_ inclusions are absorbed and precipitated within the slag phase.

The matrix phase is mainly composed of γ-TiAl. In addition, the magnified XRD pattern of the primary TiAl peak (Figure 14f) reveals a slight shift toward a lower diffraction angle. This occurs because nitrogen atoms diffuse into the TiAl lattice, forming a Ti-Al-N solid solution and resulting in lattice distortion [37].

After casting, deposits were found on the inner wall of the graphite crucible. These deposits were collected and analyzed to reveal the crucible erosion behavior and the role of carbon in the alloy.

**Figure 15:**
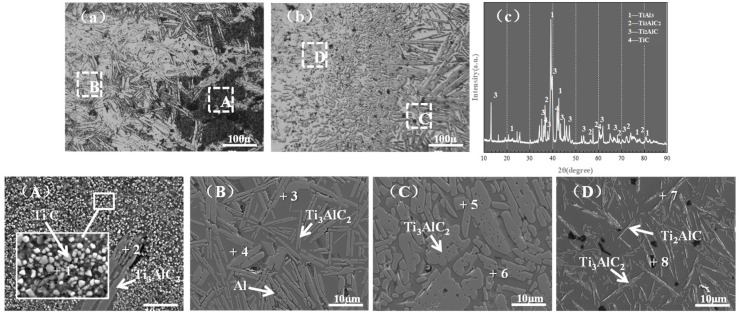
SEM and XRD analyses at the interface: (**a**) outer interface; (**b**) inner interface; (**c**) XRD pattern at the interface; Regions (**A**–**D**).

The SEM and XRD characterizations of the deposits attached to the inner wall of the crucible are presented in Figure 15. Table 7 summarizes the EDS compositional analysis at various points marked in Figure 15.

Figure 15a shows the outer interface of the graphite crucible, where a large number of irregular, bright, near-spherical particles are densely distributed. According to the EDS and XRD analyses, these particles are the TiC phase. This is because the C atoms in the graphite crucible are extremely active at high temperatures and diffuse from the crucible wall to the melt region near the wall. At this point, the Ti atoms in the melt react with C atoms to form TiC. With increases in heating temperature and time, atomic transport between C atoms in the graphite crucible and the alloy melt is intensified, and more TiC is formed at the crucible wall [38]. TiC has an extremely high melting point and is stable in the experimental temperature range of 1600–1750 °C, resulting in the adhesion of TiC on the graphite crucible wall.

Figure 15b shows the inner interface of the graphite crucible, where a large number of lath-shaped substances are randomly distributed in the inner layer of the TiC layer. Figure 15c shows the inner layer of the B region, where a large number of massive or coarse lath-shaped second phases are distributed. EDS analysis shows that the second phases in the B and C regions have the same composition, which is identified as the Ti_3_AlC_2_ phase via XRD analysis. The Ti_3_AlC_2_ phase is formed through the peritectic reaction between liquid TiAl and solid TiC (Equation (7)):

2TiC + TiAl→Ti_3_AlC_2_ (7)

During the cooling process after casting, the adhered products in the inner layer experience significant undercooling and solidify rapidly, preventing sufficient phase precipitation, thus forming a massive or coarse lath-shaped morphology. The B region is adjacent to the graphite crucible, and the slow heat dissipation rate during cooling results in a small undercooling, which provides sufficient time for the precipitation and grain growth of the Ti_3_AlC_2_ phase, so the phase precipitates completely and presents a complete lath-shaped morphology. Point 4 in Figure 15b indicates the Al phase, with 85.63 wt.% Al determined via EDS analysis. This phase forms due to the formation of a large number of carbides through the reaction of C and Ti in the C region, which consumes a large number of Ti atoms and leads to a large amount of Al segregation between the lamellar colonies.

Figure 15D shows the matrix region adjacent to the interface, where the second phase is fine lath-shaped or acicular, which is different from the B and C regions. Combined with EDS and XRD analyses, this phase is identified as the Ti_2_AlC phase [39], which is formed through the phase transformation of Ti_3_AlC_2_ in the C region (Equation (8)):

Ti_3_AlC_2_ → TiC + Ti_2_AlC (8)

The phase transformation temperature of Ti_2_AlC is higher than that of Ti_3_AlC_2_ (FactSage8.3), and the higher temperature in the D region (near the heated melt) can promote this phase transformation, so the Ti_2_AlC phase is considered to form before casting.

While the carbon pick-up in the final VAR ingot was strictly controlled to 0.085 wt.%, the severe interfacial interaction and the formation of carbides (TiC, Ti_3_AlC_2_, and Ti_2_AlC) highlight a significant challenge for industrial scalability. For continuous, large-scale production, the progressive erosion of the graphite crucible would inevitably exacerbate carbon contamination and reduce the crucible lifespan. Therefore, alternative crucible strategies must be implemented for future industrial deployment. One highly viable industrial solution is induction skull melting (ISM) utilizing a water-cooled segmented copper crucible, which relies on a solidified alloy ‘skull’ to contain the melt, thereby completely eliminating exogenous carbon and ceramic contamination. Alternatively, applying highly thermodynamically stable refractory coatings, such as yttria (Y_2_O_3_) or barium zirconate (BaZrO_3_) [40,41,42], could effectively serve as a barrier to prevent melt–crucible interactions. Transitioning to these advanced crucible technologies will be a critical step toward further reducing the interstitial carbon content for aerospace-grade applications.

**Figure 16:**
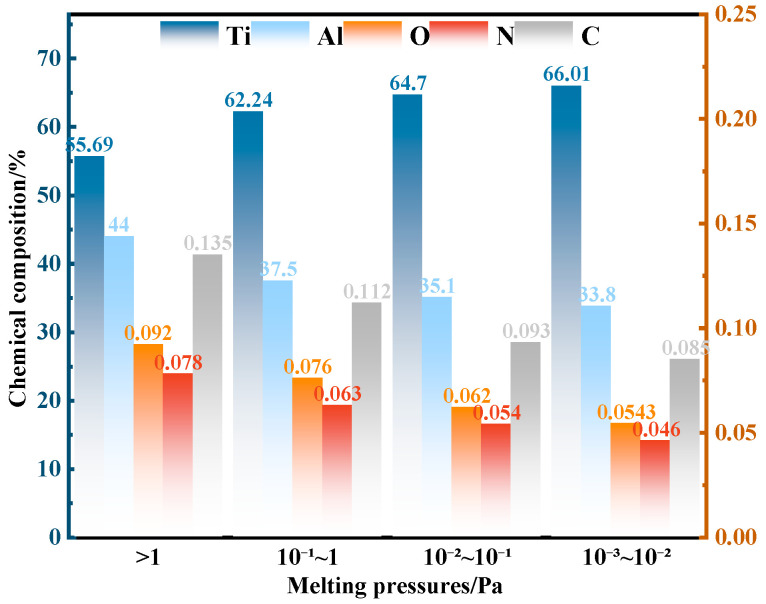
Variation in Ti, Al, O, N, and C contents (wt.%) in TiAl alloys as a function of chamber pressure.

**Figure 17:**
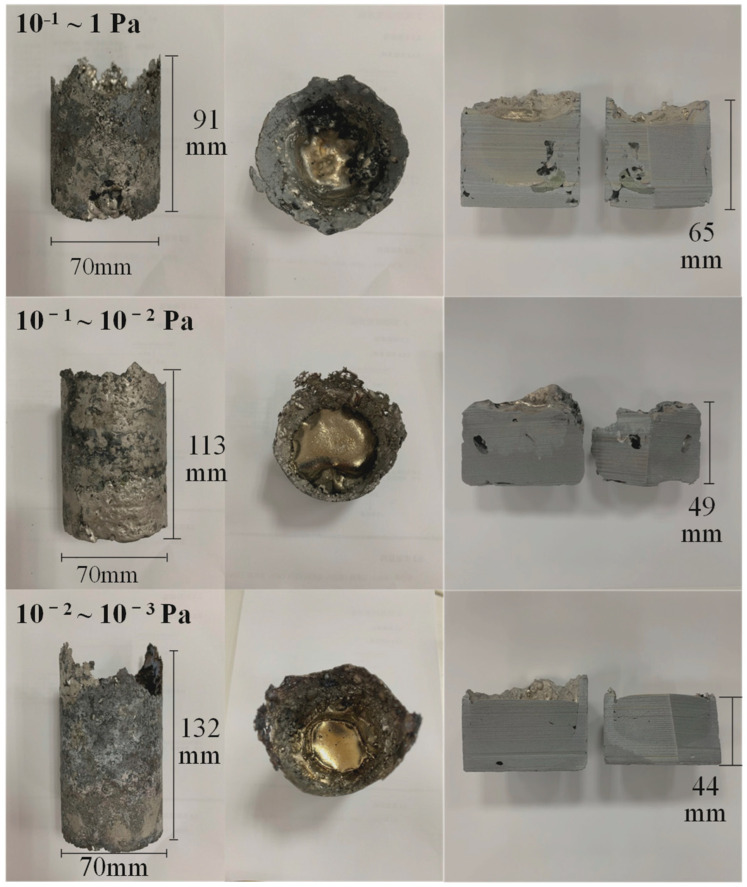
Macroscopic morphology of consumable products under different melting pressures.

Figure 16 presents the variation in the chemical composition (wt.%) of the alloy under different chamber pressures, while Figure 17 shows the appearance of the alloy ingots. With decreasing chamber pressure and increasing vacuum level, degassing and purification effects are significantly enhanced, leading to a gradual reduction in the contents of interstitial impurity elements (O, N, and C). Meanwhile, the Al content decreases markedly from 44 wt.% to 33.8 wt.% (corresponding to approximately 47.6 at.%) due to severe vacuum volatilization, which confirms that Al is prone to evaporation during the VAR process, as discussed above. Furthermore, lowering the chamber pressure effectively eliminates shrinkage porosity and significantly improves the densification and structural integrity of the final alloy ingots [43].

**Figure 18:**
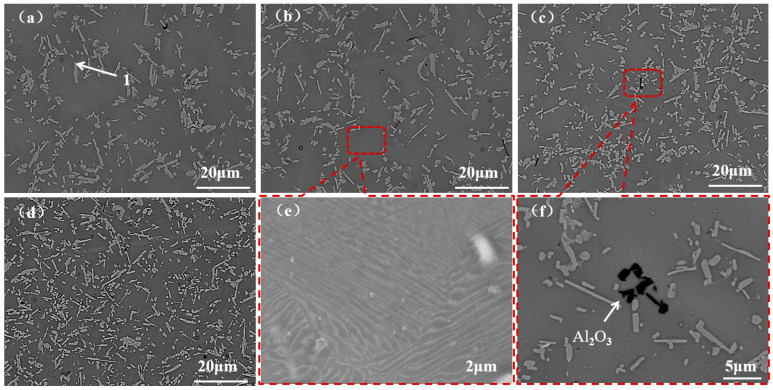
SEM images of alloys under different melting pressures: (**a**) >1 Pa; (**b**) 10^−1^–1 Pa; (**c**) 10^−2^–10^−1^ Pa; (**d**) 10^−3^–10^−2^ Pa; (**e**) magnified matrix region; (**f**) inclusion morphology.

Figure 18 shows SEM images of the alloy under different VAR vacuum pressures. After VAR treatment, the alloy is composed of the matrix phase and the lath-shaped second phase, and the numbers of slag-phase and Al_2_O_3_ inclusions in the field of view are extremely low. The second phase in the alloy is the α_2_ phase (Point 1 in Figure 18a), and the number of α_2_-phase inclusions increases with the increase in the vacuum degree (decrease in pressure), with refined grains and more uniform distribution. Combined with Figure 17, the increase in the vacuum degree leads to the decrease in the Al content and the increase in the Ti content in the alloy, which drives the alloy to transform into Ti-rich phases. Figure 18e shows a magnified image of the matrix phase, which presents an alternating lamellar morphology with no fixed orientation between the lamellar colonies, belonging to the nearly lamellar structure composed of γ and α_2_ phases. This microstructural evolution is thermodynamically highly consistent with the optimized final composition of the VAR ingot (~47.6 at.% Al), which strictly falls within the classical γ + α_2_ dual-phase region required for the formation of a nearly lamellar structure.

Figure 18f shows the morphology of the inclusions in the refined alloy, which are all Al_2_O_3_, with a size of 0–3 μm, and distributed as aggregates. This is because the large inclusions are broken into fine particles by the high-energy arc and become trapped in the matrix during the subsequent rapid cooling process.

**Figure 19:**
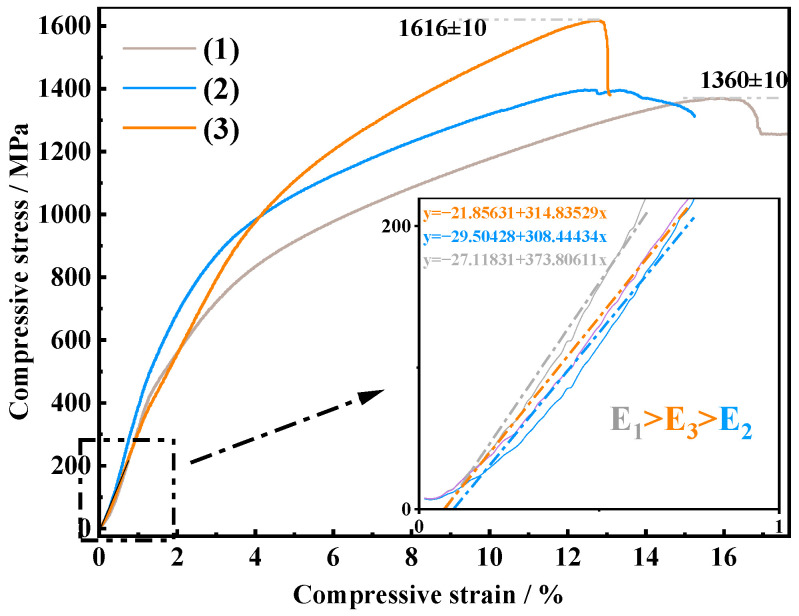
Compressive stress–strain curves of the alloy under different processes: (1) SHS primary ingot; (2) electrode rod; (3) VAR alloy.

Figure 19 shows the room-temperature compressive stress–strain curves of TiAl alloys prepared via the three processes [44]. It should be noted that since the alloys were in the as-cast state without subsequent thermomechanical processing, they exhibited inherent room-temperature brittleness, making valid tensile testing highly challenging. Thus, compression testing was utilized primarily as a comparative tool to evaluate the relative structural integrity and matrix strength across different processing stages. As shown in Figure 19, the VAR alloy (3) achieves the highest peak compressive strength of 1616 MPa, while the SHS primary ingot (1) exhibits a lower peak strength (about 1360 MPa) but sustains a larger plastic deformation.

Rather than being solely dictated by interstitial elements, this mechanical evolution is governed by the synergistic effects of inclusion refinement, microstructural optimization, and interstitial tuning. Firstly, the SHS primary ingot contains massive, irregular inclusions that act as severe stress concentrators, promoting premature failure. Through the coupled process, the inclusion population is drastically reduced and their size is refined to 0–3 μm, effectively mitigating defect-induced failure and elevating the overall strength [45]. The microstructural transition plays a critical role. The VAR process drives the phase fraction toward a dense, nearly lamellar γ + α_2_ structure. These abundant interphase boundaries significantly hinder dislocation motion, providing substantial strengthening. The interstitial elements contribute via solid-solution effects. The deep removal of oxygen relieves excessive lattice distortion, while the slight retention of nitrogen dissolved in the matrix further restricts dislocation slip, contributing to high strength. Therefore, the mechanical properties are synergistically controlled by the coupled optimization of inclusions, the microstructure, and interstitial elements [46].

## 4. Conclusions

This work systematically investigated a novel coupled process of SHS aluminothermic reduction, slag washing refining, and VAR for the preparation of low-interstitial γ-TiAl alloys directly from TiO_2_ raw material. The effects of key process parameters and operating conditions on alloy composition, phase structure, and inclusion evolution were clarified, and the underlying mechanisms of impurity and inclusion removal were revealed. The main conclusions are summarized as follows:

1. Thermodynamic calculations indicated that the CaO-Al_2_O_3_-SiO_2_-CaF_2_ quaternary slag system possessed the lowest melting temperature and suitable density, making it the optimal flux for γ-TiAl slag washing refining. Calcium could spontaneously reduce titanium oxides in the range of 1000–2200 K and acted as an effective deoxidizer. AlN could be fully decomposed under a vacuum of 10^−1^ Pa, while TiN could be partially decomposed under the transient high temperature of the VAR arc at 10^−2^ Pa, enabling deep denitrification of the alloy.

2. During slag washing refining, the mixture was induction-melted and held for 5 min followed by rapid casting: 1.5 wt.% Ca addition enabled efficient deoxidation, 1750 °C casting improved slag–metal separation, and a flux dosage of ≤150 g per 1000 g alloy yielded a sound microstructure. For VAR of 5 kg charge, melting was conducted at 600–900 A and 28–38 V for 8–10 min, and a vacuum pressure below 10^−2^ Pa achieved deep interstitial purification and microstructure homogenization. Meanwhile, moderate Al volatilization under high vacuum further optimized the alloy composition to the target γ-TiAl ratio.

3. The coupled SHS–slag washing–VAR process successfully produced low- interstitial γ-TiAl alloys with a composition of Ti 66.01 ± 0.5 wt.%, Al 33.8 ± 0.5 wt.%, O 0.054 ± 0.002 wt.%, N 0.046 ± 0.005 wt.%, and C 0.085 ± 0.008 wt.%, which aligns with the desired microstructure of γ-TiAl. Compared with SHS primary ingots, the final alloy showed a remarkably reduced number of inclusions, with the size refined to 0–3 μm and uniform distribution in the matrix.

Future work will focus on optimizing crucible technologies (e.g., induction skull melting) to further restrict carbon pick-up and applying thermomechanical processing to evaluate the tensile ductility of the as-cast ingots.

## Figures and Tables

**Table 1:** Experimental reagents and their manufacturers.

Reagents	Grade	Manufacturer
CaO	AR	Tianjin Zhiyuan Chemical Reagents Co., Ltd., Tianjin, China
Al	99.00%	CITIC Jinzhou Metal Co., Ltd., Jinzhou, China
TiO_2_	99.00%	Jinzhou Pengda Titanium Dioxide Manufacturing Co., Ltd., Jinzhou, China
KClO_3_	AR	Xilong Science Co., Ltd., Guangdong, China
MgO	AR	Tianjin Zhiyuan Chemical Reagents Co., Ltd., Tianjin, China
SiO_2_	AR	Tianjin Zhiyuan Chemical Reagents Co., Ltd., Tianjin, China
CaF_2_	AR	Tianjin Zhiyuan Chemical Reagents Co., Ltd., Tianjin, China
Al_2_O_3_	AR	Tianjin Zhiyuan Chemical Reagents Co., Ltd., Tianjin, China
Ar	99.99 vol.%	Shenyang Kejin Chemical Gas Co., Ltd., Shenyang, China

**Table 2:** Raw material ratio for SHS-prepared TiAl primary ingots (wt.%).

Raw Materials	TiO_2_	Al	CaO	KClO_3_
Mass ratio (wt.%)	33.46	30.59	20.65	15.30

**Table 3:** Optimization parameters of the density model (T_ref_ = 1773 K).

α [cm^3^/(mol·K)]	*V_i_* (cm^3^/mol)				
0.0014	Al_2_O_3_	CaF_2_	CaO	SiO_2_	MgO
	39.33	24.55	18.68	25.75	11.94

**Table 4:** Possible reactions of Ca with titanium oxides.

No.	Chemical Reaction
1	3TiO_2_ + Ca(l) = Ti_3_O_5_ + CaO
2	TiO_2_ + Ca(l) = TiO + CaO
3	1/2TiO_2_ + Ca(l) = 1/2Ti + CaO
4	1/2Ti_3_O_5_ + Ca(l) = 3/2TiO + CaO
5	1/5Ti_3_O_5_ + Ca(l) = 3/5Ti + CaO
6	TiO + Ca(l) = Ti + CaO

**Table 5:** Thermal decomposition reactions of oxides and nitrides in the γ-TiAl alloy.

Chemical Reaction
Al_2_O_3_ = 2Al + 3/2O_2_
TiO = Ti + 1/2O_2_
TiO_2_ = Ti + O_2_
SiO_2_ = Si + O_2_
AlN = Al + 1/2N_2_
TiN = Ti + 1/2N_2_

**Table 6:** EDS component analysis at different points in Figure 14 (wt.%).

Point	Ti	Al	O
a	66.81	33.19	-
b	71.01	28.99	-
c	2.99	79.17	17.84
d	2.73	79.00	18.27
e	68.19	31.81	-
f	66.59	33.41	-
g	3.23	79.43	17.34
h	67.43	32.57	-
i	84.66	15.34	-
j	2.85	78.62	18.54
k	3.60	76.52	19.88
l	3.13	78.93	17.94

**Table 7:** EDS component analysis at different points in Figure 15 (wt.%).

Point	Ti	Al	C	N	O	Phase
1	60.48	9.12	23.62	2.82	3.96	TiC
2	69.98	14.71	15	0.01	0.29	Ti_3_AlC_2_
3	69.9	14.69	15.07	0.13	0.2	Ti_3_AlC_2_
4	1.54	85.63	8.03	1.83	2.96	Al
5	70.02	14.89	14.68	0.24	0.17	Ti_3_AlC_2_
6	70.99	14.33	14.23	0.3	0.15	Ti_3_AlC_2_
7	68.35	17.21	11.44	0.42	2.58	Ti_2_AlC
8	74.12	12.49	8.21	0.11	5.07	Ti_2_AlC

## Data Availability

The original contributions presented in this study are included in the article. Further inquiries can be directed to the corresponding authors.
